# Quality Assessment of Websites Providing Information on Human Papillomavirus Vaccines in Thailand

**DOI:** 10.31557/APJCP.2019.20.11.3473

**Published:** 2019

**Authors:** Naratassapol Likitdee, Nampet Jampathong, Chumnan Kietpeerakool

**Affiliations:** *Department of Obstetrics and Gynaecology, Faculty of Medicine, Khon Kaen University, Khon Kaen, Thailand. *

**Keywords:** Human papillomavirus, vaccines, quality assessment, websites

## Abstract

**Objectives:**

To assess the quality of websites that provide information about human papillomavirus (HPV) vaccination using the criteria laid out by the World Health Organization Global Advisory Committee on Vaccine Safety.

**Methods:**

This study assessed the quality of 154 Thai-language websites accessible in November 2018 via the three most popular search engines. Differences in scores were reported as mean difference (MD) and 95% confidence interval (CI).

**Results:**

With regard to credibility, all of the websites examined indicated their mission but, in most cases (91.6%), lacked transparency with regard to sponsorship. In terms of content, almost all of the websites (97.4%) met our writing/editing and content accuracy standards but did not provide complete information regarding the benefits of vaccination or adverse events associated with it. None of the websites contained information regarding their editorial/review process. All of the websites were accessible and were designed to be adaptable to mobile device screens. News and personal websites had lower credit scores than those of academic institutions (MD -0.63, 95% CI -1.05 to -0.20; MD -0.71, 95% CI -1.16 to -0.25, respectively).

**Conclusion:**

Most of the websites met standards in terms of writing/editing and content accuracy. However, fundamental information regarding the benefits and adverse events associated with HPV vaccination were infrequently reported, the editorial process and transparency issues were rarely addressed.

## Introduction

Websites have become a common source of healthcare information for the general population. The majority of the internet users report to use the internet for their health purpose i.e. searching health information related to their hospital visits, the potential side-effects of medication, and complications of a particular treatment (Diaz et al., 2002; Flynn et al., 2006; Andreassen et al., 2007). Medical websites, therefore, can influence readers’ beliefs and decisions regarding medical treatment. However, the quality of information provide in the websites varies widely (McMullan, 2006).

Several strategies for assessing the quality of the websites providing healthcare information have been proposed in several previous studies (Craigie et al., 2002; Hargrave et al., 2006; Tozzi et al., 2010; Irwin et al., 2011, Rosen et al., 2018). The World Health Organization (WHO) has concerned the quality of the website in providing health information which can influence the success of health promotion strategies, particularly with regard to vaccination programs. In response to this issue, the Global Advisory Committee on Vaccine Safety (GACVS) has launched a tool for assessing the quality of websites that contain information related to vaccination, which aims to assist people in finding those websites that contain accurate information (WHO, 2018).

Vaccination against human papillomavirus (HPV) infection is effective in the primary prevention of cervical cancer and has been incorporated into many countries’ national vaccination programs (Lowy et al., 2008). Over 10 years of monitoring, HPV vaccine is concluded to be a safe and effective intervention (Centers for Disease Control and Prevention, 2019). However, various misconceptions about HPV vaccination exist, resulting in many people have opted to remain unvaccinated (McCave, 2010; Albright and Allen, 2018). The educational interventions to improve HPV knowledge e.g. the internet resources form national organization is the one of the methods to increase HPV awareness and HPV vaccination rates (Hughes et al., 2009; Leung et al., 2019). A previous study suggested that information on the internet not only affected peoples’ knowledge, beliefs, and acceptance regarding the HPV vaccine but also affected the physicians’ intention to recommend HPV vaccines (Riedesel et al., 2005; McRee et al., 2012). However, the quality of information regarding HPV vaccination information has been found to widely vary among various websites (Tozzi et al., 2010). This study was conducted to assess the quality of Thai-language websites that provide information about HPV vaccination by applying the GACVS good information practices for vaccine safety web sites as a tool for evaluation.

## Materials and Methods

**Figure 1:**
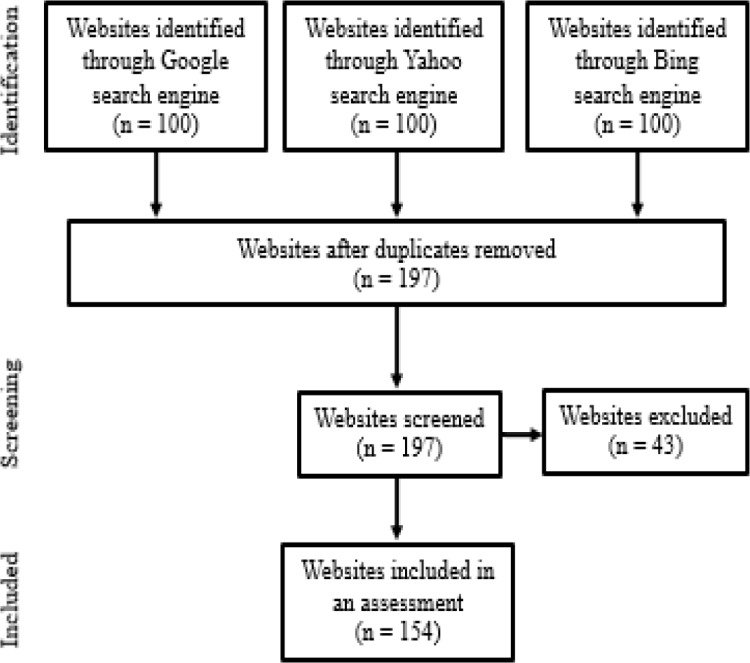
Search Strategy

We reviewed the first 100 Thai websites providing information about the HPV vaccination to appear in search results of the 3 most popular search engines in Thailand; Google, Yahoo, and Bing (StatCounter Global Stats, 2018). We excluded duplicate websites and those that were not accessible from the assessment. The selection process is summarized in Figure 1. We used the following search parameters: “HPV Vaccine” (waksin aet phi wi; HPV vaccine) OR “HPV vaccine” OR “human papillomavirus vaccination,” and restricted the search to websites displayed in the Thai language. The search was conducted on November 1, 2018. We included in our analysis the first 100 websites to appear in the results from each search engine. After duplicate websites were removed, the details of the included websites were independently reviewed by two authors (NL and CK). Websites were categorized as News, Portal, Personal, Medical center/hospital/public health agency/university, or pharmaceutical/commercial company websites. The tone of each website was categorized as neutral, positive, negative, or ambiguous. 

We assessed the quality of websites according to the following criteria laid out by the WHO GACVS: credibility, the accuracy of the content, accessibility, and web design.

Issues that need to be addressed to determine credibility are the mission of the website, disclosure of ownership/sources, transparency of sponsorship, accountability to users, data protection and privacy, and responsible partnering. In terms of content, the authority of sources, the date information was last updated, the independence of the editorial/review process, standards of writing/editing, the accuracy of content, and completeness need to be evaluated. Accessibility is judged based on consistency of the website’s availability, its adaptability to mobile devices, presence of large and unnecessary graphics, instructions for downloading pdf files (if available), presence of “printer-friendly” and “share” buttons on each page, accessibility of the home page from other pages, presence of broken links, availability of technical support for users, and presence of information on the legality or distribution of material. Design quality was determined based on whether the website was deemed pleasant and professional. A website received one point for each satisfied criterion. The maximum scores for credibility, content, accessibility, and design were 6, 6, 10, and 1, respectively. Disagreements between the two reviewers were solved by consensus-based discussion. 

Frequencies and descriptive statistics were calculated and presented as numbers and percentages. Mean scores were compared among different types of websites using one-way ANOVA and reported as mean difference (MD) and associated 95% confidence interval (CI). Post-hoc pairwise comparisons of the assessment scores of medical center/hospital/public health/university sites and other types of the website were performed using Dunnett’s test. R version 3.5.3 was used in the analysis.

## Results

The selection process of websites to be included in this study is summarized in Figure 1. The forty-three websites were excluded secondary to a lack of relevant information (25) and inaccessible links. The characteristics of the 154 websites are displayed in Table 1. The greatest number of websites were the medical center, hospital, public health agency, and university sites, followed by news and personal websites. Nearly 80 percent of the websites exhibited a positive tone with regard to the HPV vaccine, while only 2.6 percent were negative. Most of the websites did not link to other sites, but most of those that did link to public health or university websites.

Table 2 shows the assessment scores of 154 websites stratified by each specific criterion. In terms of the credibility criteria, all websites indicated their mission, and 149 (96.8%) disclosed their ownership/sources. The majority of websites (91.6%) were not transparent in terms of sponsorship.

With regard to content, 150 of the websites (97.4%) met our standards of writing/editing, and their contents were deemed accurate and up-to-date. However, they did not provide complete information regarding the benefits and adverse events of the vaccine. None of the websites provided information about their editorial/review process. One hundred and eight of the websites (70.1%) were judged as having incomplete content. 

In terms of accessibility, all websites were consistently available, and their design was adaptable to mobile device screens. There were 110 websites (71.4%) that provided buttons that would allow users to share content via social media or email. Almost none (14.3%) provided links to a printer-friendly version. Only 5 websites (3.3%) provided pdf files for download, and of those, only 3 provided instructions on how to download these files. 

Regarding website design, 148 of the websites (96.1%) were determined to be pleasant and professional. The remaining six (3.9%) were deemed unpleasant due to it being difficult to read the content being presented.

Credibility, content, and accessibility assessment scores cross-tabulated by type of website are displayed in Table 3. The medical center/hospital/public health agency/university sites had the highest mean credibility score, while the personal and news sites had the lowest. The news sites had the highest mean scores in content and accessibility, whilst the personal sites had the lowest. 

Pairwise comparison of each type of site using the medical center/hospital/public health/university websites as a reference is displayed in Table 4. News, portal, and personal websites had significantly lower mean credibility scores than medical center/hospital/public health agency/university websites. News sites had significantly better content and accessibility scores than medical center/hospital /public health/university websites.

**Table 1:** Characteristics of the 154 Retrieved Websites

Characteristics	Total (N=154)	%
Type		
News	37	24.0
Portal sites	17	11.0
Personal sites	29	18.8
Medical centers/hospitals/public health agencies/ universities	43	27.9
Pharmaceutical/commercial companies	28	18.2
Tone		
Neutral	28	18.2
Positive	119	77.3
Negative	4	2.6
Ambiguous	3	1.9
Links to other websites*		
Medical center/hospital	5	3.2
Public health agency/university pages	18	11.7
Pharmaceutical/commercial companies	3	1.9
No links	120	77.9
Others	12	7.8

*Websites may have linked more than one website

**Table 2:** Credibility, Content, Accessibility and Design of 154 Websites

	Yes		No		Cannot evaluate	
	n	(%)	n	(%)	n	(%)
Credibility						
Mission of site	154	100.0	0	0.0	0	0.0
Disclosure of ownership/source	149	96.8	5	3.2	0	0.0
Transparency of sponsorship	13	8.4	141	91.6	0	0.0
Accountability to users	120	77.9	34	22.1	0	0.0
Data protection and privacy	9	5.8	145	94.2	0	0.0
Responsible partnering	36	23.4	118	76.6	0	0.0
Content						
Authority of sources	109	70.8	45	29.2	0	0.0
Currency	114	74.0	40	26.0	0	0.0
Independence of the editorial/review process	0	0.0	154	100.0	0	0.0
Standards of writing/editing	150	97.4	4	2.6	0	0.0
Content accurate and current	150	97.4	3	2.0	1	0.6
Completeness (benefits, harms and side effects)	45	29.2	108	70.1	1	0.7
Accessibility						
Consistency of availability	154	100.0	0	0.0	0	0.0
Web design allows for adaptability to mobile devices	154	100.0	0	0.0	0	0.0
Lack of large and unnecessary graphics	149	96.8	5	3.2	0	0.0
Presence of instructions to download pdf files (if available)	3	2.0	2	1.3	149	96.7
Links to printer-friendly versions of each page	22	14.3	132	85.7	0	0.0
“Share” buttons (social media, email)	110	71.4	44	28.6	0	0.0
Easy access to the home page from any page	112	72.7	42	27.3	0	0.0
No broken links	151	98.1	1	0.6	2	1.3
Availability of technical support	122	79.2	31	20.1	1	0.7
Availability of information on legality or distribution of material	101	65.6	52	33.7	1	0.7
Design						
Pleasant and professional design	148	96.1	6	3.9	0	0.0

One point was given for each criterion that was met. The maximum scores were 6 for credibility, 6 for content,10 for accessibility, and 1 for web design.

**Table 3:** Score Distribution in the Domains of Credibility, Content, and Accessibility by Type of Website

Domain	All	News	Portal sites	Personal sites	Academic agencies*	Pharmaceutical/commercial companies	P-value
Credibility; mean (SD)	3.1 (0.8)	2.8 (0.6)	3.2 (0.9)	2.8 (0.7)	3.5 (0.7)	3.3 (0.9)	0.0049
Content; mean (SD)	3.7 (0.9)	4.0 (0.4)	3.9 (0.9)	3.8 (1.1)	3.3 (1.0)	3.6 (1.0)	0.0204
Accessibility; mean (SD)	7.0 (1.1)	7.5 (0.8)	7.4 (1.1)	6.6 (1.1)	6.7 (1.0)	7.0 (1.1)	0.0186

*Including medical centers, hospitals, public health agencies, and universities

**Table 4:** Pairwise Comparisons Using Websites Hosted by Academic Agencies as a Reference

Domain	News vs academic agencies*		Portal sites vs academic agencies*		Personal sites vs academic agencies*		Pharmaceutical/commercial companies sites vs academic agencies*	
	MD	95 % CI	MD	95 % CI	MD	95 % CI	MD	95 % CI
Credibility	-0.63	-1.05,-0.20	-0.23	-0.77,0.31	-0.71	-1.16,-0.25	-0.18	-0.64,0.28
Content	0.62	0.11,1.13	0.53	-0.12,1.18	0.44	-0.10,0.99	0.26	-0.29,0.81
Accessibility	0.82	0.24,1.39	0.66	-0.08,1.39	-0.15	-0.76,0.47	0.34	-0.29,0.96

*Including medical centers, hospitals, public health agencies, and universities; MD, mean difference; CI, confidence interval

## Discussion

Two authors independently reviewed the included websites using the criteria proposed by the WHO GACVS in their “good information practices for vaccine safety web sites” as a benchmark. The majority of the websites (97.4%) included in this study met the writing/editing and content-accuracy standards. Fundamental information that needed to be addressed in the websites, including benefits and adverse events associated with HPV vaccination, the website’s editorial/ review process, and potential conflicts of interest in terms of sponsorship, were infrequently reported. News, portal, and personal websites had significantly lower mean credibility scores than the medical center/hospital/public health agency/university websites.

Websites providing health-related information can be different depending on the particular purpose of the website developer. In this study, the majority of websites were launched by the medical centers, hospitals, public health agencies, and universities. From the survey of websites that provided information related to HPV vaccination in Italian and English, most retrieved websites (33.5%) were from private agencies (Tozzi et al., 2010).

We also found that the quality of a website was related to the agency responsible for its hosting and development. This is consistent with the results of previous studies, which found that websites developed by medical centers, hospitals, public health agencies, and universities had higher quality than websites hosted by the private agencies (Hargrave et al., 2006; Tozzi et al., 2010; Irwin et al., 2011). However, that news, portal, and personal websites were more likely than those hosted by academic agencies to be easy to access. This highlights the need for academic agencies to improve the accessibility of their websites in order to allow users better access to the highly credible information contained therein.

The perception of potential harm caused by HPV vaccination is amongst the leading causes of people refusing or delaying HPV vaccination (McCave, 2010; Gilkey et al., 2017; Albright and Allen, 2018) Websites should thus provide comprehensive information regarding the benefits and potential risks of HPV vaccination based on reliable evidence. However, almost none of the websites examined in our study did so, meaning that efforts should be made to improve these sites in this regard. 

Declaration of sponsorship and transparency of the editorial and review process of these websites is also important information for readers. Sponsored websites may be biased in favor of the sponsors’ interests, and an inappropriate editorial review process of the content of a website may result in misleading information. Information regarding sponsorship and the editorial/review process was rarely disclosed in the websites we examined. The majority (91.6%) did not declare their sponsors, and none provided information regarding their editorial/review process.

There are various approaches that can be used to determine the quality of websites (Craigie et al., 2002; Hargrave et al., 2006; Tozzi et al., 2010; Irwin et al., 2011, Rosen et al., 2018). This study applied the criteria proposed by the WHO GACVS as a tool for evaluating the quality of the retrieved websites, which is an internationally accepted standard for assessing the quality of websites that provide information related to vaccination. Our assessment of Thai-language websites with regard to these criteria revealed several issues that should be addressed and areas for improvement. Because of the cross-sectional data assessment employed in this study, it was not possible to determine changes in the quality of these websites over time. 

In conclusion, websites developed and hosted by academic agencies, including medical centers, hospitals, public health agencies, and universities, were deemed highly credible and thus important information sources of information regarding HPV vaccination for Thai-language speakers. Improvements in the accessibility of these websites, however, are needed. Issues regarding the transparency of sponsorship and the editorial/review process should also be addressed.
